# What Are the Place and Modalities of Surgical Management for Pancreatic Neuroendocrine Neoplasms? A Narrative Review

**DOI:** 10.3390/cancers13235954

**Published:** 2021-11-26

**Authors:** Samuel Frey, Eric Mirallié, Maëlle Le Bras, Nicolas Regenet

**Affiliations:** 1 Université de Nantes, Quai de Tourville, 44000 Nantes, France; samuel.frey@chu-nantes.fr (S.F.); eric.mirallie@chu-nantes.fr (E.M.); 2 L’institut du Thorax, Université de Nantes, CNRS, INSERM, CHU de Nantes, 44000 Nantes, France; 3 Chirurgie Cancérologique, Digestive et Endocrinienne, Institut des Maladies de l’Appareil Digestif, CHU de Nantes, 44000 Nantes, France; 4 Endocrinologie, Diabétologie et Nutrition, L’institut du Thorax, CHU Nantes, 44000 Nantes, France; maelle.lebras@chu-nantes.fr

**Keywords:** neuroendocrine neoplasms, pancreatic tumors, pancreatic surgery

## Abstract

**Simple Summary:**

Although pancreatic neuroendocrine neoplasms represent less than 5% of all pancreatic cancers, their incidence rate has risen dramatically over the last decade, mainly due to improved detection methods. They are considered malignant by default. However, their outcomes are variable depending on their presentation in the onset of hereditary syndromes, hormonal secretion, grading, and extension. Therefore, although surgical treatment has long been suggested as the only treatment of pancreatic neuroendocrine neoplasms, its modalities are an evolving landscape, especially since parenchyma-sparring pancreatectomy and endoscopic approaches instead of large pancreatic resections have been proposed. Moreover, in selected cases, watchful strategies are on balance with surgical resection, but the accurate size cut-off of the tumor remains to be established. The aim of this narrative review is to describe the current recommended surgical management for pancreatic NENs and controversies in light of the actual recommendations and recent literature.

**Abstract:**

Pancreatic neuroendocrine neoplasms (panNENs) are a heterogeneous group of tumors derived from cells with neuroendocrine differentiation. They are considered malignant by default. However, their outcomes are variable depending on their presentation in the onset of hereditary syndromes, hormonal secretion, grading, and extension. Therefore, although surgical treatment has long been suggested as the only treatment of pancreatic neuroendocrine neoplasms, its modalities are an evolving landscape. For selected patients (small, localized, non-functional panNENs), a “wait and see” strategy is suggested, as it is in the setting of multiple neuroendocrine neoplasia type 1, but the accurate size cut-off remains to be established. Parenchyma-sparring pancreatectomy, aiming to limit pancreatic insufficiency, are also emerging procedures, which place beyond the treatment of insulinomas and small non-functional panNENs (in association with lymph node picking) remains to be clarified. Furthermore, giving the fact that the liver is generally the only metastatic site, surgery keeps a place of choice alongside medical therapies in the treatment of metastatic disease, but its modalities and extensions are still a matter of debate. This narrative review aims to describe the current recommended surgical management for pancreatic NENs and controversies in light of the actual recommendations and recent literature.

## 1. Introduction

Neuroendocrine neoplasms (NENs) are rare tumors derived from cells with neuroendocrine differentiation, predominantly found in the lung, the digestive tract and the pancreas [1]. Pancreatic NENs (panNENs) represent less than 5% of all pancreatic cancers [2], and 12.1% of all NENs [3]. Their incidence rate has risen significantly during the past 40 years [4,5], mainly due to increased diagnosis of localized and low grade panNENs, which could suggest that improved detection methods and awareness of the disease play a major role in this phenomenon [5,6].

Although regrouped under a common appellation, panNENs in fact represent a heterogeneous group of neoplasms with various prognoses. Indeed, depending on the presence of hormonal secretion, which can lead to specific complications, their occurrence in the setting of hereditary syndromes, their degree of differentiation, grade, and extensions, the management of panNENs greatly differs and represents an evolving landscape and a therapeutic challenge [7,8].

Because systemic treatments seem to only stabilize the disease because of inherent or acquired drug resistance and poor delivery within the pancreas [9,10], surgery remains a cornerstone of the management of panNENs and remains the only curative treatment [11,12,13], with its indications and modalities being influenced by the aforementioned heterogeneity of these tumors. Several consensuses have been proposed to describe the surgical treatment of panNENs, including the North American Neuroendocrine Tumor Society (NANETS) [14], the National Comprehensive Cancer Network (NCCN) [15], and the European NeuroEndocrine Tumor Society (ENETS) [16]. However, controversies exist between these recommendations and emerge from the recent literature.

The aim of this narrative review is to describe the current recommended surgical management for panNENs and controversie, in light of the actual recommendations and recent literature.

## 2. Presentation, Diagnosis and Pre-Operative Workup

### 2.1. Clinical Presentation

Based on the presence of well-defined clinical symptoms related to hormonal secretion, panNENs are separated into two major groups: functioning (F-panNENs) and non-functioning panNENs (NF-panNENs). The four major F-panNENs causing specific syndromes are insulinomas (secretion of insulin leading to hypoglycemia), gastrinomas causing Zollinger Ellison syndrome (gastrin leading to recurrent ulcer disease), glucagonomas (glucagon causing necrotic migratory erythema, undernutrition and diabetes mellitus) and VIPomas causing Werner Morrison syndrome (vasoactive intestinal peptide leading to water diarrhea, hypokalemia and achlorhydria) [17]. In contrast with F-panNENs, NF-panNENs do not cause specific symptoms other than those related to tumor burden but are frequently incidentally diagnosed on imagery before their occurrence [18].

### 2.2. Biochemical Analysis

Serum biomarkers are frequently used for the diagnosis workup of panNENs. Chromogranin A has been described for the diagnosis of NF-panNENs, with a sensitivity ranging between 66–73% and a low specificity between 10 and 35% [19,20,21]. As it is associated with a significant propensity to cause false-positive results in the presence of numerous benign and malignant conditions, this marker could be a more suitable marker during follow-up in selected patients [22,23]. Other peptides, including neuron specific enolase (NSE), progastrin releasing peptide (PRP) and pancreatic polypeptide (PP), have been proposed as diagnosis markers of NF-panNENs, though they are associated with variables sensitivity and specificity [22]. The diagnosis of F-panNENs includes specific biological testing adapted to the symptoms caused by hormonal hypersecretion. For example, when symptoms suggest organic hypoglycemia, the diagnosis of insulinoma will be retained in the case of elevated levels of insulin, pro-insulin and C-peptide after 72 h fasting [24].

### 2.3. Imaging

Abdominal imaging is a major step in the diagnosis and pre-therapeutic workup of panNENs. Both abdominal CT-scan and MRI have demonstrated their performances to detect the primitive tumor, CT-scan being associated with 82% sensitivity and 96% specificity for panNENs and MRI with 79% sensitivity and 76% specificity [25]. However, CT-scan is believed to describe major vessel involvement that determines tumor resectability (see below) with more accuracy than MRI and should be performed systematically [16]. On the other hand, MRI detects liver metastases with high accuracy, and is used to measure their burden when their resection is envisaged [26,27]. Moreover, MR cholangiopancreatography, as well as endoscopic ultrasound, are of special interest to estimate the relation between the tumor and the main pancreatic duct and are highly recommended when enucleation is envisaged (i.e., mainly insulinomas or NF-panNENs < 2 cm, see below) [16,28].

In addition, the use of functional imaging using radiolabeled somatostatin analogs is systematically recommended to assess liver metastases and extra-abdominal disease [16,29] except for insulinoma, in which a lower amount of somatostatin receptors is present [30]. For panNENs, positron emission tomography with CT with ^68^Ga labeled somatostatin analogs has been shown to have the best sensitivity and specificity (92% and 83%, respectively) and is the exam of choice [25], which has been suggested to improve staging in comparison with conventional imaging [31]. Of note, poorly differentiated panNENs, which have a higher proliferation rate, are better evaluated using ^18^F-fluorodeoxyglucose-positron emission tomography [32]. Invasive methods such as arterial stimulation venous sampling can be performed in addition to imaging exams when the latter do not allow for the localization of insulinomas or glucagonomas [33].

## 3. Grading and Staging of Pancreatic Neuroendocrine Neoplasms

All NENs are considered malignant by default [34]. A three-grade classification has been adopted to describe NENs, as proposed by the World Health Organization (WHO) classifications for panNENs [35] and the 2018 International Agency for Research on Cancer (IARC) and WHO consensus [36]. This grading is based on proliferation markers that are the Ki-67 cell labelling index and the mitotic count (number of mitoses/mm²): G1 for low grade, G2 for intermediate grade and G3 for high grade NENs [35,36]. Poorly differentiated panNENs, namely neuroendocrine carcinomas, are associated with the worst prognosis [37] and are excluded from this grading since they are consistently high grade. The current classification and grading, as described by the 2017/2019 WHO classification and IARC, is proposed in Table 1. Grading of panNENs when a surgical management is envisaged is often a necessity, since tumor grade can influence the surgical indication and especially is predictive of lymph node metastasis (LNM) in a recent meta-analysis [38].

PanNENs staging is currently performed using two main systems that are mostly similar, namely the modified ENETS staging classification [39] and the 8th edition of the AJCC Cancer Staging Manual [40], which are summarized in Table 2. Local extension mainly depends on peripancreatic vessel involvement: superior mesenteric vein, superior mesenteric artery, coeliac axis and common hepatic artery. Encasement of one of those arteries or superior mesenteric vein thrombosis means that the panNEN is considered locally advanced [16].

## 4. Surgical Management of Localized Pancreatic Neuroendocrine Neoplasms (Stage IA–IIB)

### 4.1. Surgical Management for Localized NF-panNENs

#### 4.1.1. Surgical Indications for Localized NF-panNENs

Surgery is the standard treatment for localized NF-panNENs larger than 2 cm [15,16]. However, tumors with size <2 cm, which represent more than 20% of all NF-panNENs [41], are believed to be less aggressive [42]. Therefore, an active surveillance has been proposed and is now on balance with surgical resection for small localized and asymptomatic NF-panNENs [15,16]. However, in daily practice this “wait and see” approach seems to be poorly accepted by both patients and clinicians and is currently a subject of controversy [43].

Several reports have compared active surveillance with surgical management for small NF-panNENs; however, all of these were small and retrospective and none of the mean/median follow ups exceeded five years. Disease-related mortality, tumor growth, occurrence of metastasis and secondary surgical intervention are the main outcomes reported in the current literature. Active surveillance seemed not to increase disease-related mortality in comparison with initial surgical resection [44,45,46,47,48]. In one study comparing 56 patients under surveillance with 193 who were operated on, the latter group presented with a better survival (10 years survival 82.6% versus 53.7% with observation, *p* < 0.001) [49]. However, several patients presented with metastasis initially (25% and 7% in the observational and surgical groups, respectively), and patients with syndromic panNENs were also included (7 with VHL and 23 with MEN1). Moreover, in this study, only 38 patients in the observation group presented with an initial tumor size <2 cm and multivariate analysis showed that surgery was not associated with improved survival for tumors with an initial size <1.5 cm [49].

Numerous studies have reported no modification in tumor size [44,50,51] or a percentage of patients displaying a significant increase in tumor size between 2% (3/145 patients) and 4.5% (2/44 patients) [46,48] when active surveillance was chosen for small tumors. However, in another report, which used a 3 cm cut-off to define small tumor, 7.7% of patients (8/104) underwent surgery because of tumor growth during surveillance [47].

Metastasis did not occur during follow-up for small tumors [44,48] or were not different when compared with operated patients. In a French multicenter study, 14% of the patients with observations and 11% of the operated patients presented with metastasis during follow-up [50]. In another report that included 15 patients under surveillance and 20 who underwent surgery, metastasis occurred in 3 patients during observation, but all had initial tumor size >2 cm [51].

Two meta-analyses reported that secondary surgical resection after a period of observation was necessary in 12–14% of patients with NF-panNENs < 2 cm [43,52]. Tumor growth has been suggested to be the only objective parameter to address the patient to surgery [43,45,46,47,48], but patient preference is to be taken into account and was the cause of a secondary resection in many studies [46,47].

Despite the favorable outcomes resulting in an observational approach as described in the aforementioned study, one subject of controversy is the definition of small NF-panNENs. Indeed, the choice of a 2 cm cut-off has been made on the belief that tumors with size <2 cm were less aggressive [42]. However, in a large retrospective study including 1854 patients with NF-panNENs < 2 cm, LNM was present in 29% and distant metastases in 10% of patients [53], which is supported by a recent meta-analysis [38]. Furthermore, one could consider that the difficulty in diagnosing LNM on imaging, which strongly impacts survival, could favor the surgical indication. Others reported the occurrence of distant metastasis in 5–10% of such patients [54,55]. In a recent multicenter retrospective study including 80 patients with NF-panNENs, we proposed that the best cut-off to discriminate between an indolent and an aggressive tumor was 1.7 cm (OR 10.8 [95%CI 1.17–53.2], *p* = 0.03) [50]. Another study suggested that only tumors >1.5 cm could have a survival benefit with surgery [49]. Further investigations are strongly needed to clearly identify the optimal cut-off that is balanced with patient comorbidity and risk of postoperative death, life expectancy, tumor location, and risk of post-operative complications (fistula and morbidity) [52]. The question of how the tumor size should be measured, i.e., by endoscopic ultrasound or morphologic imaging, also deserves to be answered.

When chosen, a “wait and see” strategy should be reserved to selected patients [16]. As no long-term data are available to demonstrate the safety of this approach, which should be evaluated with at least 10 years of follow-up because of the slow progression of panNENs [43,52]. Therefore, old patients and patients with comorbidities and high surgical risk are likely to be good candidates [56]. Surgery should also be performed in patients with main pancreatic duct involvement, which is known to be an independent predictor for aggressiveness [57]. Tumors of the pancreatic head are also less likely to undergo surveillance because they could be associated with a higher risk of LNM [58].

The place of an extensive workup to ascertain the diagnosis before choosing a watchful strategy is still a matter of debate. EUS-guided biopsy by fine needle aspiration was recommended by the authors of [45], aiming to rule out lesions mimicking panNENs such as accessory spleen or cystadenoma or renal cell carcinoma metastasis. On the other hand, others argue that cytology could be associated with a high risk of false positive finding and should be avoided [56]. By extension, whether the grade of small panNENs should be obtained by biopsy fine needle aspiration using the Ki-67 index is still controversial. One can argue that tumors of higher grade (G2 and G3) should be resected given the higher risk of aggressiveness. However, even though Ki-67 index measurement on biopsy by fine needle aspiration had excellent accuracy in determining the tumor grade [48], obtaining a sufficient amount of tissue material is often not possible [45,59]. Other studies should be performed to ascertain the role of tumor grading before choosing a watchful strategy.

No current guidelines describe the optimal imaging follow-up when the watchful attempt is chosen. Repeating axial imaging and assessing serum biomarker levels (chromogranin A) every 6 months seems to be a reasonable option [22].

#### 4.1.2. Extent of Surgery in Localized NF-panNENs

Classic surgical resections, including pancreaticoduodenectomy and left pancreatectomy, are usually proposed for localized NF-panNENs. However, these procedures are associated with a significant amount of pancreatic endocrine and exocrine insufficiency (9–60% and 7–35%, respectively) [60,61,62], which deserve to be taken into account in the context of panNENs, as these patients display excellent long-term prognosis [60]. Therefore, parenchyma sparing procedures such as enucleation or central pancreatectomy have been proposed for NF-panNENs, limiting endocrine and exocrine insufficiency (2.5–7.5%, 0–18%, respectively) [63,64,65], but the rate of post-operative complications, especially fistula, is suggested to be higher than in classic pancreatectomy [66]. In this line, a recent large propensity-matching study including 109 patients with panNENs per group showed a higher incidence of pancreatic fistula after enucleation (24.5%) compared with pancreaticoduodenectomy and distal pancreatectomy (14.0%, *p* = 0.049).

In its latest recommendations, the ENETS group stated that, given the risk of inadequate surgical margin clearance and absence of lymphadenectomy, such a parenchyma-sparring approach should be reserved for small tumors <2 cm and insulinoma [16]. In this line, a recent report suggested that R1 resection margin was more frequent with enucleation than classic pancreatectomy for NF-panNENs in a cohort, including both large (>2 cm) and small tumors [67]. On the other hand, another report including 130 patients with panNENs (of all size, 85% non-functioning) found no differences in terms of overall morbidity between classic and parenchyma sparing pancreatectomy, and a shorter hospital stay after parenchyma sparing pancreatectomy [68]. However, in this report, small low-grade panNENs were more likely to undergo parenchyma-sparing procedures, inducing an inclusion bias. Therefore, for instance, classic pancreatectomy seems accurate for NF-panNENs >2 cm until the role and modalities of parenchyma-sparing procedure for their management is better defined. On the other hand, parenchyma sparing procedures seem to be safe for small tumors, as reported by Falconi et al. who reported no mortality and only 8% recurrence in a retrospective study including 50 patients with small NF-panNENs (41 with tumor size <2 cm) [69], therefore, the parenchyma sparring procedure is the procedure of choice for small panNENs when surgery is the chosen approach. In this case, the distance to the main pancreatic duct should be taken into account to decrease the risk of post-operative fistula [50].

LNM has been suggested to be a critical prognostic factor for panNENs [70,71,72], especially when tumor size is <4 cm [73]. They are more frequent in panNENs >2 cm [54] and localized in the pancreatic head [72,74]; however, small tumors of the body/tail also have a 20% estimated probability of developing LNM [72]. These findings strongly suggest that regional lymph node dissection should systematically complete pancreatic resection for tumors >2 cm, which is the recommended approach [15,16]. However, a recent propensity score weighted analysis including 2664 panNENs by Mao et al. reported no significant improvement of overall survival after lymph node dissection, even for tumors >2 cm [75], and raises the question of selecting patients suitable for lymph node dissection.

**Figure 1:**
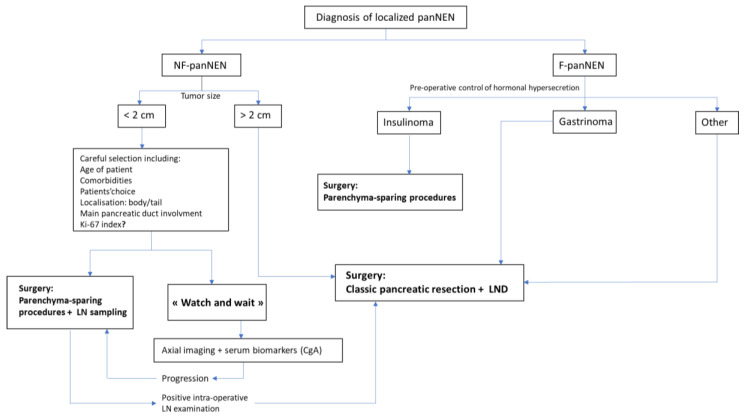
Surgical management for sporadic localized NF-panNENs (Stage IA–IIB). F/NF-panNENs: Functional/non-functional pancreatic neuroendocrine neoplasms, LN: lymph node, LND: lymph node dissection, CgA: chromogranin A.

In the case of small NF-panNENs which undergo parenchyma sparring procedures, the risk of understaging the tumor in some patients in the absence of lymph node examination should lead to systematically performing lymph node sampling when enucleation or central pancreatectomy are performed and a positive intra-operative examination should lead to classic pancreatectomy [69]. Figure 1 summarizes surgical management for sporadic NF-panNENs.

### 4.2. Surgical Management of Localized F-panNENs

The goal of the surgical management of localized F-panNENs has for objectives the control of clinical symptoms as well as the prevention of tumor growth and the occurrence of metastasis [76]. Before surgery, the control of hormonal hypersecretion is necessary for insulinoma, gastrinoma, and VIPoma to avoid potential life-threatening complications [60].

#### 4.2.1. Surgery for Sporadic Insulinomas

Surgery for sporadic insulinoma is associated with a cure rate superior to 90% in numerous reports [59,77,78], in accordance with a previous systematic review showing a 93% cure rate and a 7.2% recurrence rate [79]. Therefore, surgery should be envisaged for all patients with resectable sporadic insulinoma [29]. Since insulinomas are mostly benign tumors, pancreatic-sparing procedures are preferred, enucleation being the procedure of choice when the distance between the tumor and the main pancreatic duct is superior to 2–3 mm [60]. The laparoscopic approach can be proposed as a feasible and safe procedure without impairing oncologic outcomes [80,81,82], with a 2014 meta-analysis reporting reduced hospital stay (weighted mean difference −5.64 [95% confidence interval −7.11–−4.16], *p* < 0.00001), without difference in operative times, post-operative mortality and overall morbidity [83]. As therapeutic alternatives, endoscopic approaches involving ultrasound-guided radiofrequency ablation and ethanol injection are emerging and can also be considered [84,85,86]. Glucose metabolism recovery after resection of insulinoma seems to occur in most of the cases in a recent retrospective study including 77 patients treated with enucleation, with only one patient presenting with chronic diabetes mellitus requiring treatment [87]; however, blood glucose should be monitored carefully during hospitalization and after discharge, as a few patients need small doses of insulin for a period of several days or weeks [79].

#### 4.2.2. Surgery for Sporadic Gastrinomas

Surgery for gastrinoma has been a controversial issue, given the efficacy of the medical therapy to treat Zollinger Elisson syndrome [88] and the fact that the 10-year biochemical disease-free survival is achieved only in about 20–45% of patients [76,89]. Although gastric hypersecretion has been found to persist in 62% of patients with a mean 8 years despite normogastrinemia in a prospective study [90], others demonstrated that a significant proportion of disease-free patients are able to decrease or stop all antisecretory drugs after surgery [91]. Furthermore, a study comparing 160 operated versus 35 unoperated patients with gastrinoma (17% pancreatic only) showed that patients in the surgical group developed fewer liver metastases (5% vs. 29%, *p* = 0.0002) and had a lower disease-related death rate during follow up (1% vs. 23%, *p* < 0.00001, mean follow-up 19.1 years) [92]. Furthermore, a 2012 prospective study on 58 patients with Zollinger-Ellison syndrome with negative pre-operative imaging showed that experimented surgeon could find the tumor per-operatively in 98% of cases [93]. Therefore, all patients with localized sporadic gastrinoma should undergo surgery. Ninety percent of gastrinomas involve the duodenum or the pancreatic head and will undergo pancreaticoduodenectomy, otherwise a distal pancreatectomy is performed. Lymph node dissection is always necessary, as a retrospective study of 48 patients with sporadic gastrinoma (18 localized in the pancreas, 12 with synchronous liver metastasis) showed that systematic lymph node dissection is associated with surgery resulting in a higher initial cure rate (100% vs. 64% without systematic lymphadenectomy, *p* = 0.017) and a lower death rate related to the disease (0% versus 30%, *p* = 0.037) [94].

The other F-panNENs, such as somatostatinomas or VIPomas are rarely diagnosed before the occurrence of metastasis, whose resectability will decide the management (see below). Whenever possible, R0 resection with lymphadenectomy is the option of choice [60,76]. The surgical management of sporadic localized F-panNENs is summarized in Figure 1.

### 4.3. Specificities for Surgery in Syndromic panNENs

#### 4.3.1. Surgery for panNENs in the Context of Multiple Endocrine Neoplasia

Multiple endocrine neoplasia type 1 (MEN1) is an autosomal dominant syndrome related to a mutation of the *MEN1* gene [95] and is characterized by a lifetime risk of developing primary hyperparathyroidism, duodenopancreatic NENs and pituitary tumors [96]. PanNENs’ penetrance in MEN1 reaches 60–100% for NF-panNENs and more than 50% for F-panNENs [8,97]. Early onset, high rate of aggressiveness and multiplicity of tumors including multiple small endocrine tumors referred to as “microadenomatosis” characterize the panNENs associated with MEN1 [97,98].

Surgery is to be envisaged with caution for NF-panNENs in these patients, as they present a high risk of developing new panNENs within the pancreatic remnant (63% of the case with a median follow-up of 109 (range 1–264) months [99]). Moreover, when analyzing 108 patients, Triponez et al. showed a correlation between tumor size, metastasis occurrence and survival in the context of MEN1-related NF-panNENs [100], suggesting that small tumors are indolent. Therefore, several reports demonstrated the benefits of a watchful strategy for NF-panNENs < 2 cm in the context of MEN1 [101,102], which is a well-accepted strategy unlike that for sporadic NF-panNENs, especially as somatostatin analogues are strongly suggested to be safe and effective in this case [103]. In their recent prospective study, Triponez et al. showed that active surveillance was associated with a low risk of disease-specific mortality, with stable disease after a mean of 10.3 +/− 3.5 years follow-up in 60.9% of patients [104]. When surgery is performed, the parenchyma-sparring procedure is recommended to prevent endocrine and exocrine pancreatic functions and preserve quality of life [105], with reoperations often being necessary and not associated with increased morbidity when performed in expert centers [106].

Surgery for MEN1-related insulinoma (4–8% of all insulinoma [107]) is believed to be associated with good outcomes [108,109], especially in terms of hypoglycemia cure rate [108,110,111]. Enucleation appears to be the best option for patients with solitary or dominant tumors, distal pancreatectomy associated with enucleation of tumors in the head could be proposed in the case of multiple tumors [110], in accordance with the current recommendations [105]. However, 30% of patients being diagnosed with a unique tumor on MRI, CT or EUS appear to have multiple resections during surgery, underscoring the fact that multiple tumors should always be searched intra-operatively and patients should be aware that aggressive resection could be performed [110].

F-panNENs related with MEN1 are mostly represented by gastrinomas (10–54% of patients with gastrinoma [8]). However, almost all of these gastrinomas occurs in the duodenum, pancreatic gastrinoma in the setting of MEN1 being very rare [112]. The surgical indication for MEN1-related gastrinoma is a controversial issue [91,105]. On one hand, small tumors are thought to have an excellent prognosis even without surgery, they frequently present with lymph node and/or liver metastasis, and surgery exerts a more moderate effect on hypergastrinemia than sporadic gastrinoma [8,91,105,112]. Furthermore, MEN1-related gastrinomas are believed to be highly responsive to somatostatin analogue therapies [113]. On the other hand, reports suggest that aggressive surgery could be beneficial for duodenal/pancreatic gastrinoma in the context of MEN1 [114], even in the case of locally advanced disease [115]. Therefore, no recommendation exists concerning the surgical treatment of MEN1-related gastrinoma, and potential benefits of surgical indication should be weighted with the risk of an aggressive surgery and potential reoperations.

Of note, cases of NF-panNENs and gastrinomas have been described in the setting of multiple neuroendocrine neoplasia type 4, a recently described syndrome due to the mutation of the *CDKN1B* gene. The management of these tumors is comparable to that in MEN1 [116].

#### 4.3.2. Surgery for panNENs in the Context of Von Hipple Lindau Disease

Von Hipple Lindau disease (VHL) is an autosomal dominant syndrome caused by a mutation of the *VHL* tumor suppressor gene, inducing various benign and malignant tumors of the central nervous system, renal carcinomas or cysts, pheochromocytomas, epididymal cystadenoma and pancreatic tumors and cysts [117]. PanNENs are reported in 15% of patients with VHL, are almost exclusively non-functional [8,118,119], and are multiple in more than 50% of the cases [120]. They are believed to be less aggressive than sporadic panNENs [120].

Data regarding surgical indications for VHL-associated NF-panNENs are limited. Tumor size is the strongest risk factor of aggressiveness. In a prospective study enrolling 108 patients with VHL-related panNENs, tumors with size >3 cm were more likely to develop metastasis (24 versus 3.6%, *p* < 0.005) [121]. In a recent retrospective report including 17 VHL-related panNENs, seven patients with tumor size <3 cm were treated conservatively, with all but one displaying stable disease at a 2 year median follow-up [122]. The current trend is to propose a conservative treatment for NF-panNENs with size <3 cm in the body/tail of the pancreas, this cut-off being decreased to 2 cm in the pancreatic head when suitable for enucleation [118,123]. Tumors with increasing size during follow-up, associated with a germline mutation in exon 3 and suspicion of LNM (if 90% of the primary tumor seems resectable), are discussed surgical indications [118,121,123,124]. As shown in a retrospective study including 11 patients with small VHL-associated panNENs, tumors with size <1.5 cm do not progress when left in place [125] and therefore should not be removed when identified intra-operatively [118].

#### 4.3.3. Surgery for panNENs in the Context of Other Hereditary Syndromes

Type 1 neurofibromatosis (NF-1) is due to a mutation of the *NF-1* gene and is identified by the presence at different extents of pigmentary skin lesions, neurofibromas, skeletal abnormalities, and brain and peripheral nerve tumors [126]. PanNENs, not being part of the classic features of NF-1, are present in fewer than 10% and are almost exclusively duodenal somatostatinomas located in the peri-ampullar region, which does not secrete hormones but frequently causes jaunice, biliary dilatation and pancreatitis [8]. Distant metastases are present in 30% of cases at diagnosis [127]. Real panNENs are exceptional in the onset of NF-1 [128]. Few reports have described the outcomes of surgery for panNENs in this context. In their review of 76 cases of periampullary tumors in the setting of NF-1 of which 38 were periampullary NENs, Relles et al. suggested that a large resection with lymph node harvest is indicated in these patients, especially when tumor size is >2 cm [129]. Other reports suggest that an adequate radical surgery should be offered whenever possible [8,130,131,132]. However, because NENs in the context of NF-1 are not an increased cause of death, especially because of their rarity [133], the authors suggested that a more conservative approach should be attempted [8].

Rarely, pancreatic NET can be found in the context of tuberous sclerosis, an autosomal dominant inherited condition being characterized by the presence of multiples hamartomas and tumor-like hamartomatous lesions, skin lesions and disabling neurological features [134]. Reported tumors are insulinomas and nonfunctional pancreatic NET, for which surgery is recommended whenever possible [8].

## 5. Surgery for Locally Advanced Diseases (Stage III)

Several studies have shown that portal or superior mesenteric vein resections and reconstructions associated with pancreatic resection for locally advanced pancreatic carcinoma were associated with acceptable morbidity and mortality and can be performed safely [135,136,137]; however, concurrent procedures are associated with poor oncologic outcomes and are considered a predictor of cancer recurrence [135,137,138]. Nevertheless, because panNENs do not generally exhibit signs of local aggressiveness, the question of surgery in the case of abutment with vessel or neighborhood organs, which occurs in approximately 20% of cases [139], deserves to be studied. Few data exist regarding this specific issue.

Only retrospective cohorts are available to evaluate the safety and efficacity of surgical resection for locally advanced panNENs, and many of them included few patients and mixed patients with metastatic diseases. Norton et al., studying 46 patients with NENs involving major vessels (30 panNENs, 12 in the duodenum, 18 also displaying liver metastasis) reported nine cases of superior mesenteric vein or portal vein reconstructions. They showed that disease-free survival was not impaired in the case of vascular reconstruction [139]. Another report on seven patients who underwent vascular reconstruction (four with initial liver metastasis) showed that only one patient without initial metastatic disease exhibited disease progression [140]. When comparing 43 patients who underwent surgical resection for advanced disease (27 with liver metastasis) with 91 patients operated for localized disease, the multivariate analysis of Birnbaum et al. showed that vascular or adjacent organ resection did not impair disease-free survival [141]. On the other hand, in another study including 95 patients who underwent pancreaticoduodenectomy, among which 26 also had organ/vascular resection, post-operative complications were more frequent (70.3 versus 26.1%, *p* < 0.001) and 5 years disease-free survival lower after additional organ resection for low-grade tumors [142]. However, in accordance with the previous studies, the authors did not find any difference of overall 5 years survival after extended pancreatectomy. In a recent study including 99 patients with non-metastatic T3/T4 panNENs who underwent vascular and/or near organ resection, Titan et al. reported a 91% five-year survival and a 35% recurrence rate, the latter being favorized by surrounding organ resection (excluding blood vessel) in multivariate analysis (HR 6.15 [95% CI, 1.61–23.55], *p* = 0.008) [143].

Overall, although the extension of the tumor to nearby organs and vessels seems clearly to be associated with a higher risk of recurrence, which is suggested to be associated with higher tumor grade [142], the acceptable five-year survival observed in these studies suggests the benefit of performing extended surgery for locally advanced panNENs. In light of this, the ENETS recommends that selected patients with low (G1) or intermediate grade (G2) could benefit from extended pancreatic resection with organ/vessel resection, provided that macroscopic complete resection can be achieved [16].

Importantly, none of these studies specifically focused on the outcomes of surgery for locally advanced F-panNENs versus NF-panNENs, although patients with NF-panNENs displaying vascular involvement on pre-operative imaging had decreased survival compared with F-panNENs in the series of Norton et al. [139]. However, a retrospective study reported that eight patients with F-panNENs who underwent en-bloc resection of adjacent organs without known metastasis were cured from their endocrinopathy [144], suggesting that the place of large resection to treat hormonal syndrome deserves to be studied.

The place of neoadjuvant therapies before surgery for locally advanced panNENs is of particular interest. Although the recent study of Xie et al. found no improvement in overall survival in patients who underwent perioperative systemic therapies (the type not being described) in comparison with patients with surgery alone for localized panNENs [145], specific pre-operative protocols have gained interest in this context. Peptide receptor radionuclide therapy [146] and more recently the use of capecitabine combined with temozolomide (CAPTEM) [147,148] have been shown to decrease the tumor burden and may facilitate surgery for these advanced tumors.

## 6. Surgery for Metastatic Diseases (Stage IV)

**Figure 2:**
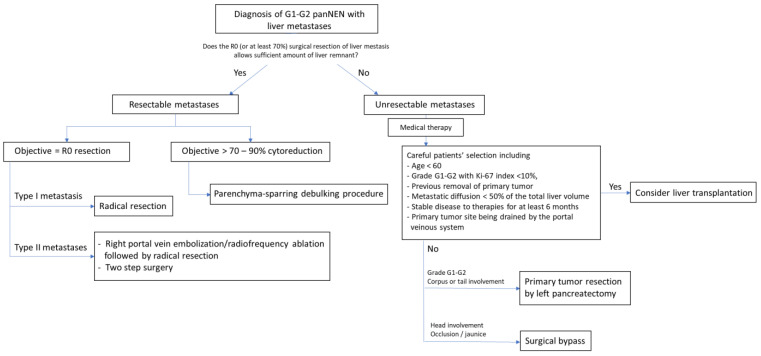
Surgical management for sporadic metastatic NF-panNENs (Stage IV). PanNEN: Pancreatic neuroendocrine neoplasm.

Being lately symptomatic, NF-panNENs present with distant metastasis at diagnosis in 60% of cases [149]. Patients with metastatic panNENs exhibit a 23-month [95%CI 20–26] median survival according to the population-based study of Yao et al., in comparison with 124 months [95%CI 80–168] for localized disease [150]. Currently, many medical options have be proposed for metastatic patients, including targeted therapy, namely everolimus and sunitibib [151,152], chemotherapy [22], or peptide receptor radionucleide therapy [153]. However, surgery maintains an important place for these patients, being the only curative treatment [154], which can benefit from the aforementioned medical options when used as a neo-adjuvant therapy. Moreover, giving the relatively indolent course of panNENs even in case of metastases and the fact that the liver is generally the only metastatic site, surgery could be an attractive option, taking place in the setting of multimodal treatment, in which the place of each therapeutic option remains to be clearly defined. The place of the surgical management in metastatic panNENs is summarized in Figure 2.

### 6.1. Surgery for Patients with Resectable Metastases

#### 6.1.1. PanNENs Liver Metastases Resection Is Associated with Improved Survival

The benefits of liver metastasis excision in terms of overall survival have been highlighted by many authors: retrospective studies including metastatic NENs (including both panNENs and NENs from other origins) reported a five-year survival rate between 59.9 and 82% [155,156,157,158,159]. A retrospective study comparing 91 patients with panNENs who had liver metastasis resection with 75 patients treated conservatively showed that the former had a better median survival (97 versus 36 months, *p* < 0.0001) [101]. In a recent report, 184 patients with adjunction of metastasis resection to primary tumor resection had an increased median overall survival compared to pancreaticoduodenectomy alone (71.8 versus 93.2 months, *p* < 0.001) [160]. Metastasectomy alone (without primary tumor resection) has also been found to increase the median overall survival in comparison with no surgery (25.2 versus 15.2 months, *p* < 0.001), which strongly suggest that aggressive surgery is an option for these patients.

On the other hand, liver metastases resection for panNENs has been associated with a low disease-free survival and a high rate of recurrence. In the study of Sarmiento et al. which included 170 patients with liver metastasis resection, the recurrence rate was 84% at 5 years [158]. Another study including 47 patients (15 with panNENs) with hepatic resection showed that the 10-year liver recurrence rate of the disease was 75% [159]. In this study, pancreatic primary site, the completeness of surgery, the presence of bilateral or more than 10 liver metastases, were correlated with the disease-free survival. In this line, Cusati et al. reported a 10.7% five-year progression-free survival for patients with R0 resection and 3.5% for R1 resection in non-functioning panNENs [157].

Although they are frequently included in the management of metastatic panNENs in the current practice, the benefit of the adjunction of neoadjuvant therapies before liver metastasis resection is not well established [161]. Among the most promising modalities, the use of neoadjuvant cytotoxic therapy including chemotherapeutic agents fluorouracil, doxorubicin, and streptozocin (FAS) in patients with synchronous liver metastases increased overall survival and recurrence-free survival in comparison with patients who underwent surgery alone [162]. The CAPTEM protocol has also been suggested to be associated with a high radiological response of liver metastases in the setting of panNENs [163] and has been proposed to facilitate the selection of patients suitable for surgical resection [164]. Peptide receptor radionuclide therapy, alone or in adjunction with CAPTEM, could also be envisaged as neoadjuvant therapy [165,166]. Further studies are clearly needed to better define the place of these treatments before liver metastases resection.

#### 6.1.2. What Should Be the Extent of Liver Resection?

The distribution profile of liver metastases from NENs have led to a three-type classification which influence the surgical resection: type I (single metastasis), type II (predominant metastatic bulk accompanied by smaller deposits) and type III (disseminated disease) [167]. ENETS recommendations actually propose type I and II metastases to be treated by radical resection (R0) [16]. However, complete resection of bilobar metastasis (type II) is limited by the risk of post-operative liver failure related to small-sized liver remnants. One-step (in association with percutaneous right portal vein embolization and/or radiofrequency ablation [168]) or two-step surgical approaches can be proposed to allow complete resection of liver metastases and limit the risk of hepatic failure. The two-step procedure consists of (i) a complete clearance of liver left-sided metastasis, the resection of the primitive tumor and a right portal vein ligation in order to induce hypertrophy of the cleared left lobe, (ii) followed by right hepatectomy eight weeks later [169]. The application of this procedure to NENs (50% panNENs) was associated with 94% five-year overall survival rate and 50% five-year disease-free survival rate [170]. Another surgical approach, namely associating liver partition and portal vein ligation for staged hepatectomy (ALPPS), has been suggested to improve liver metastasis clearance in comparison with the aforementioned conventional two-step surgical resection in patients with colorectal cancer [171]. A recent study showed that this approach allowed R0 resection in 90% of patients with liver metastases in the context of NENs; however, the low 2-year disease-free survival (41.8%) and the high rate of complications should make us consider this approach with caution in the context of NENs [172].

However, the necessity of a R0 resection of liver metastasis has been criticized. Authors suggested that patients with non-functional NENs (including 35% panNENs) had an identical overall survival whether they had R0/R1 or R2 resection [156]. The univariate analysis performed by Elias et al. in their study showed that R0, R1 or R2 status did not impact overall survival [159]. Therefore, the authors suggested that acceptable survival can be obtained with 70–90% clearance of metastasis [158,173,174]. However, obtaining 90% cytoreduction can be obtained in only a few patients, as the study of Maxwell et al. reported that 90% and 70% debulking were obtained in 38.9% and 63.9%, respectively [175]. Furthermore, this study also showed that overall survival and progression-free survival were significantly improved after 70% reduction [175], which is in accordance with the results of another team, who reported in two retrospective studies that the liver disease progression was not correlated with the amount of resected tissue over 70% cytoreduction [174,176]. Therefore, an objective of 70% cytoreduction could be a suitable objective.

Therefore, parenchyma-sparring debulking procedure, allowing positive margins, has been proposed to treat metastatic panNENs. The study of Maxwell et al. showed that patients who underwent such procedures (ablation, enucleation, wedge resections) had only 13% major complications [175]. Importantly, these procedures were associated with a 72% five-year overall survival, which is comparable with studies including major resections [159,177], suggesting their efficacity and safety. Furthermore, the liver metastases debulking procedure can also be considered in the case of metastatic insulinoma, and might facilitate the control of insulin hypersecretion [178].

#### 6.1.3. How to Select Patients Suitable for Liver Cytoreduction?

Tumors with high grade (G3) were shown to be independently associated with poorer survival after liver metastasis resection in the study of Partelli et al. (median OS: 35 vs. 97 months for low/intermediate grade, *p* < 0.0001) [179]. Therefore, patients with G3 panNENs should be excluded from liver metastasis cytoreduction.

Although extrahepatic metastasis localizations should be ruled out in the actual recommendations [16], Morgan et al. suggested that the presence of extrahepatic localizations did not alter the results of liver metastases resection [176]. Interestingly, only the presence of metastases >5 cm was associated with liver progression in multivariate analysis in this study. Other authors suggested that, regardless of the size and the number of metastases and the presence of extrahepatic disease, the possibility of obtaining > 70% clearance of liver metastases, even with positive margins, could be a sufficient criteria for patient selection [174]. It is likely that these criteria will be expanded in future.

### 6.2. Surgery for Patients with Unresectable Metastases

The exact definition of unresectable metastases in the setting of panNENs is somewhat controversial and not clearly stated in the current literature, depending on the attempted extent of resection (i.e R0 or >70%). Disseminated disease (type III metastasis) not allowing complete metastases clearance (or at least 70% clearance according to the aforementioned studies) with sufficient amount of liver remnant to avoid liver deficiency, despite the use of multistep procedures, could be the definition of unresectable metastases.

#### 6.2.1. The Place of Primitive panNEN Resection when Unresectable Liver Metastases Are Present

Several studies reported that palliative primary tumor resection is associated with improved survival. Indeed, retrospective studies including NF-panNENs reported a longer median survival when patients underwent an excision of their primitive in comparison with conservative treatment (5.42 versus 0.83 years, *p* < 0.0001 [180] and 3.5 versus 1.0 years, *p* < 0.001 [149]). Similar improvement seems to be observed in cohorts including both functioning and non-functioning tumors [181,182]. Of note, previous primary tumor surgery has also been associated with an improved response to peptide receptor radionucleide therapy [183]. However, as underlined by a systematic review, bias could have been introduced as a radical approach could have been proposed for patients with better performance status in comparison with conservative treatment in these retrospective studies [184].

In balance with the post-operative risk of pancreatectomy, primary tumor resection for patients with low or intermediate grade tumors (G1–G2) could be considered for left pancreatectomy [182] but not for pancreaticoduodenectomy, according to the current ENETS recommendations [16]. Symptomatic (occlusion or jaunice) tumors of the head can be treated by endoscopic resection or surgical bypass [16].

#### 6.2.2. The Place of Liver Transplantation for Unresectable Liver Metastases

As a last resort for highly selected patients, liver transplantation can be envisaged in the case of unresectable metastatic disease [185]. Several retrospective studies, including metastatic NENs from all origins, suggested that liver transplantation was achieved with an overall five-year survival between 47 and 52% [186,187]. Although a pancreatic origin of the metastases has been associated with a higher risk of recurrence (27% with a mean follow-up of 15 months [188]) and poor prognosis in multivariate analysis [187], liver transplantation was associated with acceptable outcomes in a systematic review of 89 patients (69 panNENs) reporting cumulative one-, three-, and five-year survival of 71%, 55%, and 44%, respectively [189]. Criteria for patients’ selection actually includes age <60, grade G1–G2 with Ki-67 index <10%, and previous removal of primary tumor, metastatic diffusion <50% of the total liver volume and stable disease to therapies for at least 6 months [16,190], with primary tumor site being drained by the portal veinous system also being proposed by some authors [185,190].

Given multiple surgical possibilities, especially two-step procedures, allowing total resection of disseminated metastases and increasing evidence that partial metastases clearance could be sufficient to obtain acceptable outcomes, the place of liver transplantation in the setting of panNENs metastasis is yet to be determined.

## 7. Conclusions

Surgery for panNENs is a therapeutic challenge. Because of their heterogeneity, surgeons should be well aware of each tumor’s specificities, which not only drives surgical indications but also surgical modalities. This is only possible with a multidisciplinary approach, with an accurate surgical management also requiring a precise pretherapeutic workup. Current controversies include the benefit of a “wait and see” strategy for small NF-panNENs, which is accepted in the setting of MEN1, but an accurate size cut-off remains to be established. The balance between the benefits and risks of parenchyma-sparring procedures for large NF-panNENs, which could avoid pancreatic insufficiencies, also deserve to be extensively studied. Moreover, the benefits of an aggressive surgery for locally extended tumors, as well as the place and extension of liver metastases cytoreduction, need to be clarified. Caution should be applied when interpreting the current data, as they mostly result from observational studies. Controlled studies are strongly needed to provide data with a higher level of evidence and help future recommendations to decipher these controversial issues.

## Figures and Tables

**Table 1:** PanNENs classification based on WHO 2010 and IARC 2018.

Type of Tumor	Family	Differentiation	Type	Grade	Ki-67 (≥500 Cells)	Mitotic Count (/2mm²)
Pancreatic neuroendocrine neoplasms	Neuroendocrine tumors	Well differentiated	Pancreatic neuroendocrine tumors	G1 (low)	<3	<2
				G2 (intermediate)	3–20	2–20
				G3 (high)	>20	>20
	Neuroendocrine carcinomas	Poorly differentiated	Small cell type	High	>20	>20
			Large cell type			

**Table 2:** Staging system according to the modified ENETS and AJCC 8th staging classifications [39,40].

	Staging						T, N and M Definitions		
	mENETS			AJCC 8th Classification				mENETs	8th AJCC Classification
Stage	T	N	M	T	N	M	T1	Tumor limited to pancreas, <2 cm	Maximum tumor diameter ≤2 cm
IA	T1	N0	M0	T1	N0	M0	T2	Tumor limited to pancreas, 2–4 cm	Maximum tumor diameter >2 cm but ≤ 4 cm
IB	T2	N0	M0	T2	N0	M0	T3	Tumor limited to pancreas, >4 cm or invading the duodenum or common bile duct	Maximum tumor diameter >4 cm
IIA	T3	N0	M0	T3	N0	M0	T4	Tumor invades adjacent structures	Tumor involves the celiac axis or the superior mesenteric artery
IIB	T1–3	N1	M0	T1-3	N1	M0	N0	No regional lymph node metastasis	
							N1	Regional lymph node metastasis	Metastasis in 1–3 regional lymph nodes
III	T4	Any N	M0	Any T or T4	N2 or any N	M0	N2	-	Metastasis in ≥4 regional lymph nodes
							M0	No distant metastasis	
IV	Any T	Any N	M1	Any T	Any N	M1	M1	Distant metastasis	

## References

[1] Leoncini E., Boffetta P., Shafir M., Aleksovska K., Boccia S., Rindi G. (2017). Increased incidence trend of low-grade and high-grade neuroendocrine neoplasms. Endocrine.

[2] O’Grady H.L., Conlon K.C. (2008). Pancreatic neuroendocrine tumours. Eur. J. Surg. Oncol..

[3] Frilling A., Åkerström G., Falconi M., Pavel M., Ramos J., Kidd M., Modlin I.M. (2012). Neuroendocrine tumor disease: An evolving landscape. Endocr.-Relat. Cancer.

[4] Fraenkel M., Kim M., Faggiano A., De Herder W.W., Valk G.D., Knowledge NETwork (2013). Incidence of gastroenteropancreatic neuroendocrine tumours: A systematic review of the literature. Endocr.-Relat. Cancer.

[5] Dasari A., Shen C., Halperin D.M., Zhao B., Zhou S., Xu Y., Shih T., Yao J.C. (2017). Trends in the Incidence, Prevalence, and Survival Outcomes in Patients with Neuroendocrine Tumors in the United States. JAMA Oncol..

[6] Young K., Iyer R., Morganstein D., Chau I., Cunningham D., Starling N. (2015). Pancreatic neuroendocrine tumors: A review. Future Oncol..

[7] Gao L., Natov N.S., Daly K.P., Masud F., Chaudhry S., Sterling M.J., Saif M.W. (2018). An update on the management of pancreatic neuroendocrine tumors. Anti Cancer Drugs.

[8] Jensen R.T., Berna M.J., Bingham D.B., Norton J.A. (2008). Inherited pancreatic endocrine tumor syndromes: Advances in molecular pathogenesis, diagnosis, management, and controversies. Cancer.

[9] Mpilla G., Philip P.A., El-Rayes B., Azmi A.S. (2020). Pancreatic neuroendocrine tumors: Therapeutic challenges and research limitations. World J. Gastroenterol..

[10] Uri I., Grozinsky-Glasberg S. (2018). Current treatment strategies for patients with advanced gastroenteropancreatic neuroendocrine tumors (GEP-NETs). Clin. Diabetes Endocrinol..

[11] Kunz P.L., Reidy-Lagunes D., Anthony L.B., Bertino E.M., Brendtro K., Chan J.A., Chen H., Jensen R.T., Kim M.K., Klimstra D.S. (2013). Consensus Guidelines for the Management and Treatment of Neuroendocrine Tumors. Pancreas.

[12] Singh S., Dey C., Kennecke H., Kocha W., Maroun J., Metrakos P., Mukhtar T., Pasieka J., Rayson D., Rowsell C. (2014). Consensus Recommendations for the Diagnosis and Management of Pancreatic Neuroendocrine Tumors: Guidelines from a Canadian National Expert Group. Ann. Surg. Oncol..

[13] Falconi M., Bartsch D.K., Eriksson B., Klöppel G., Lopes J.M., Oconnor J.M., Salazar R., Taal B.G., Vullierme M.P., O’Toole D. (2012). ENETS Consensus Guidelines for the Management of Patients with Digestive Neuroendocrine Neoplasms of the Digestive System: Well-Differentiated Pancreatic Non-Functioning Tumors. Neuroendocrinology.

[14] Kulke M.H., Anthony L.B., Bushnell D.L., de Herder W.W., Goldsmith S.J., Klimstra D.S., Marx S.J., Pasieka J.L., Pommier R.F., Yao J.C. (2010). North American Neuroendocrine Tumor Society (NANETS). NANETS treatment guidelines: Well-differentiated neuroendocrine tumors of the stomach and pancreas. Pancreas.

[15] Kulke M.H., Shah M.H., Benson A.B., Bergsland E., Berlin J.D., Blaszkowsky L.S., Emerson L., Engstrom P.F., Fanta P., Giordano T. (2015). Neuroendocrine tumors, version 1.2015. J. Natl. Compr. Cancer Netw..

[16] Partelli S., Bartsch D.K., Capdevila J., Chen J., Knigge U., Niederle B., Van Dijkum E.J.M.N., Pape U.-F., Pascher A., Ramage J. (2017). ENETS Consensus Guidelines for the Standards of Care in Neuroendocrine Tumours: Surgery for Small Intestinal and Pancreatic Neuroendocrine Tumours. Neuroendocrinology.

[17] Cives M., Strosberg J.R. (2018). Gastroenteropancreatic Neuroendocrine Tumors. CA Cancer J. Clin..

[18] Birnbaum D.J., Gaujoux S., Cherif R., Dokmak S., Fuks D., Couvelard A., Vullierme M.-P., Ronot M., Ruszniewski P., Belghiti J. (2013). Sporadic nonfunctioning pancreatic neuroendocrine tumors: Prognostic significance of incidental diagnosis. Surgery.

[19] Yang X., Yang Y., Li Z., Cheng C., Yang T., Wang C., Liu L., Liu S. (2015). Diagnostic Value of Circulating Chromogranin A for Neuroendocrine Tumors: A Systematic Review and Meta-Analysis. PLoS ONE.

[20] Pulvirenti A., Rao D., Mcintyre C.A., Gonen M., Tang L.H., Klimstra D.S., Fleisher M., Ramanathan L.V., Reidy-Lagunes D., Allen P.J. (2019). Limited role of Chromogranin A as clinical biomarker for pancreatic neuroendocrine tumors. HPB.

[21] Di Giacinto P., Rota F., Rizza L., Campana D., Isidori A., Lania A., Lenzi A., Zuppi P., Baldelli R. (2018). Chromogranin A: From laboratory to clinical aspects of patients with neuroendocrine tumors. Int. J. Endocrinol..

[22] Ma Z.-Y., Gong Y.-F., Zhuang H.-K., Zhou Z.-X., Huang S.-Z., Zou Y.-P., Huang B., Sun Z.-H., Zhang C.-Z., Tang Y.-Q. (2020). Pancreatic neuroendocrine tumors: A review of serum biomarkers, staging, and management. World J. Gastroenterol..

[23] Modlin I.M., Bodei L., Kidd M. (2016). Neuroendocrine tumor biomarkers: From monoanalytes to transcripts and algorithms. Best Pr. Res. Clin. Endocrinol. Metab..

[24] Okabayashi T., Shima Y., Sumiyoshi T., Kozuki A., Ito S., Ogawa Y., Kobayashi M., Hanazaki K. (2013). Diagnosis and management of insulinoma. World J. Gastroenterol..

[25] Sundin A., Arnold R., Baudin E., Cwikla J.B., Eriksson B., Fanti S., Fazio N., Giammarile F., Hicks R.J., Kjaer A. (2017). ENETS Consensus Guidelines for the Standards of Care in Neuroendocrine Tumors: Radiological, Nuclear Medicine and Hybrid Imaging. Neuroendocrinology.

[26] Lestra T., Kanagaratnam L., Mulé S., Janvier A., Brixi H., Cadiot G., Dohan A., Hoeffel C. (2018). Measurement variability of liver metastases from neuroendocrine tumors on different magnetic resonance imaging sequences. Diagn. Interv. Imaging.

[27] D’Assignies G., Fina P., Bruno O., Vullierme M.P., Tubach F., Paradis V., Sauvanet A., Ruszniewski P., Vilgrain V. (2013). High sensitivity of diffusion-weighted MR imaging for the detection of liver metastases from neuroendocrine tumors: Comparison with T2-weighted and dynamic gadolinium-enhanced MR imaging. Radiology.

[28] Brient C., Regenet N., Sulpice L., Brunaud L., Mucci-Hennekine S., Carrère N., Milin J., Ayav A., Pradere B., Hamy A. (2012). Risk factors for postoperative pancreatic fistulization subsequent to enucleation. J. Gastrointest. Surg..

[29] Falconi M., Eriksson B., Kaltsas G., Bartsch D.K., Capdevila J., Caplin M., Kos-Kudla B., Kwekkeboom D., Rindi G., Klöppel G. (2016). ENETS Consensus Guidelines Update for the Management of Patients with Functional Pancreatic Neuroendocrine Tumors and Non-Functional Pancreatic Neuroendocrine Tumors. Neuroendocrinology.

[30] Reubi J.C. (2004). Somatostatin and Other Peptide Receptors as Tools for Tumor Diagnosis and Treatment. Neuroendocrinology.

[31] Sharma P., Arora S., Dhull V.S., Naswa N., Kumar R., Ammini A.C., Bal C. Evaluation of (68)Ga-DOTANOC PET/CT Imaging in a Large Exclusive Population of Pancreatic Neuroendocrine Tumors. https://pubmed.ncbi.nlm.nih.gov/25134801/.

[32] Squires M.H., Volkan Adsay N., Schuster D.M., Russell M.C., Cardona K., Delman K.A., Winer J.H., Altinel D., Sarmiento J.M., El-Rayes B. (2015). Octreoscan versus FDG-PET for neuroendocrine tumor staging: A biological approach. Ann. Surg. Oncol..

[33] Andreassen M., Ilett E., Wiese D., Slater E.P., Klose M., Hansen C.P., Gercke N., Langer S.W., Kjaer A., Maurer E. (2019). Surgical Management, Preoperative Tumor Localization, and Histopathology of 80 Patients Operated on for Insulinoma. J. Clin. Endocrinol. Metab..

[34] Bosman F.T., Carneiro F., Hruban R.H., Theise N.D. (2010). WHO Classification of Tumours of the Digestive System.

[35] Lloyd R.V., Osamura R.Y., Klöppel G., Rosai J. (2017). WHO Classification of Tumours of Endocrine Organs.

[36] Rindi G., Klimstra D.S., Abedi-Ardekani B., Asa S.L., Bosman F.T., Brambilla E., Busam K.J., De Krijger R.R., Dietel M., El-Naggar A.K. (2018). A common classification framework for neuroendocrine neoplasms: An International Agency for Research on Cancer (IARC) and World Health Organization (WHO) expert consensus proposal. Mod. Pathol..

[37] Vélayoudom-Céphise F.L., Duvillard P., Foucan L., Hadoux J., Chougnet C.N., Leboulleux S., Malka D., Guigay J., Goere D., Debaere T. (2013). Are G3 ENETS neuroendocrine neoplasms heterogeneous?. Endocr.-Relat. Cancer.

[38] Tanaka M., Heckler M., Mihaljevic A.L., Probst P., Klaiber U., Heger U., Schimmack S., Büchler M.W., Hackert T. (2020). Systematic Review and Metaanalysis of Lymph Node Metastases of Resected Pancreatic Neuroendocrine Tumors. Ann. Surg. Oncol..

[39] Luo G., Javed A., Strosberg J.R., Jin K., Zhang Y., Liu C., Xu J., Soares K., Weiss M.J., Zheng L. (2017). Modified staging classification for pancreatic neuroendocrine tumors on the basis of the American Joint Committee on Cancer and European Neuroendocrine Tumor Society systems. J. Clin. Oncol..

[40] Li X., Gou S., Liu Z., Ye Z., Wang C. (2018). Assessment of the American Joint Commission on Cancer 8th Edition Staging System for Patients with Pancreatic Neuroendocrine Tumors: A Surveillance, Epidemiology, and End Results analysis. Cancer Med..

[41] Kuo E.J., Salem R.R. (2013). Population-Level Analysis of Pancreatic Neuroendocrine Tumors 2 cm or Less in Size. Ann. Surg. Oncol..

[42] Bettini R., Partelli S., Boninsegna L., Capelli P., Crippa S., Pederzoli P., Scarpa A., Falconi M. (2011). Tumor size correlates with malignancy in nonfunctioning pancreatic endocrine tumor. Surgery.

[43] Partelli S., Cirocchi R., Crippa S., Cardinali L., Fendrich V., Bartsch D.K., Falconi M. (2017). Systematic review of active surveillance versus surgical management of asymptomatic small non-functioning pancreatic neuroendocrine neoplasms. J. Br. Surg..

[44] Lee L.C., Grant C.S., Salomao D.R., Fletcher J.G., Takahashi N., Fidler J.L., Levy M.J., Huebner M. (2012). Small, nonfunctioning, asymptomatic pancreatic neuroendocrine tumors (PNETs): Role for nonoperative management. Surgery.

[45] Gaujoux S., Partelli S., Maire F., D’Onofrio M., Larroque B., Tamburrino D., Sauvanet A., Falconi M., Ruszniewski P. (2013). Observational Study of Natural History of Small Sporadic Nonfunctioning Pancreatic Neuroendocrine Tumors. J. Clin. Endocrinol. Metab..

[46] Jung J.G., Lee K.T., Woo Y.S., Lee J.K., Lee K.H., Jang K.-T., Rhee J.C. (2015). Behavior of Small, Asymptomatic, Nonfunctioning Pancreatic Neuroendocrine Tumors (NF-PNETs). Medicine.

[47] Sadot E., Reidy-Lagunes D.L., Tang L.H., Do R.K.G., Gonen M., D’Angelica M.I., DeMatteo R.P., Kingham T.P., Koerkamp B.G., Untch B.R. (2015). Observation versus Resection for Small Asymptomatic Pancreatic Neuroendocrine Tumors: A Matched Case–Control Study. Ann. Surg. Oncol..

[48] Barenboim A., Lahat G., Nachmany I., Nakache R., Goykhman Y., Geva R., Osher E., Scapa E., Wolf I., Orbach L. (2019). Resection Versus Observation of Small Asymptomatic Nonfunctioning Pancreatic Neuroendocrine Tumors. J. Gastrointest. Surg..

[49] Zhang I.Y., Zhao J., Castillo C.F.-D., Braun Y., Razmdjou S., Warshaw A.L., Lillemoe K.D., Ferrone C.R. (2015). Operative Versus Nonoperative Management of Nonfunctioning Pancreatic Neuroendocrine Tumors. J. Gastrointest. Surg..

[50] Regenet N., Carrere N., Boulanger G., de Calan L., Humeau M., Arnault V., Kraimps J.-L., Mathonnet M., Pessaux P., Donatini G. (2016). Is the 2-cm size cutoff relevant for small nonfunctioning pancreatic neuroendocrine tumors: A French multicenter study. Surgery.

[51] Rosenberg A.M., Friedmann P., Del Rivero J., Libutti S.K., Laird A.M. (2016). Resection versus expectant management of small incidentally discovered nonfunctional pancreatic neuroendocrine tumors. Surgery.

[52] Sallinen V., Le Large T.Y., Galeev S., Kovalenko Z., Tieftrunk E., Araujo R., Ceyhan G.O., Gaujoux S. (2017). Surveillance strategy for small asymptomatic non-functional pancreatic neuroendocrine tumors–a systematic review and meta-analysis. HPB.

[53] Gratian L., Pura J., Dinan M., Roman S., Reed S., Sosa J.A. (2014). Impact of Extent of Surgery on Survival in Patients with Small Nonfunctional Pancreatic Neuroendocrine Tumors in the United States. Ann. Surg. Oncol..

[54] Haynes A.B., Deshpande V., Ingkakul T., Vagefi P.A., Szymonifka J., Thayer S.P., Ferrone C.R., Wargo J.A., Warshaw A.L., Fernández-del Castillo C. (2011). Implications of incidentally discovered, nonfunctioning pancreatic endocrine tumors: Short-term and long-term patient outcomes. Arch. Surg..

[55] Cherenfant J., Stocker S.J., Gage M.K., Du H., Thurow T.A., Odeleye M., Schimpke S.W., Kaul K.L., Hall C.R., Lamzabi I. (2013). Predicting aggressive behavior in nonfunctioning pancreatic neuroendocrine tumors. Surgery.

[56] Partelli S., Mazza M., Andreasi V., Muffatti F., Crippa S., Tamburrino D., Falconi M. (2019). Management of small asymptomatic nonfunctioning pancreatic neuroendocrine tumors: Limitations to apply guidelines into real life. Surgery.

[57] Nanno Y., Matsumoto I., Zen Y., Otani K., Uemura J., Toyama H., Asari S., Goto T., Ajiki T., Okano K. (2016). Pancreatic Duct Involvement in Well-Differentiated Neuroendocrine Tumors is an Independent Poor Prognostic Factor. Ann. Surg. Oncol..

[58] Lopez-Aguiar A.G., Ethun C.G., Zaidi M.Y., Rocha F.G., Poultsides G.A., Dillhoff M., Fields R.C., Idrees K., Cho C.S., Abbott D.E. (2019). The conundrum of <2-cm pancreatic neuroendocrine tumors: A preoperative risk score to predict lymph node metastases and guide surgical management. Surgery.

[59] Guo Q., Wu Y. (2014). Surgical treatment of pancreatic islet cell tumor: Report of 44 cases. Hepatogastroenterology.

[60] Hain E., Sindayigaya R., Fawaz J., Gharios J., Bouteloup G., Soyer P., Bertherat J., Prat F., Terris B., Coriat R. (2019). Surgical management of pancreatic neuroendocrine tumors: An introduction. Expert Rev. Anticancer Ther..

[61] Beger H.G., Poch B., Mayer B., Siech M. (2018). New onset of diabetes and pancreatic exocrine insufficiency after pancreaticoduodenectomy for benign and malignant tumors: A systematic review and meta-analysis of long-term results. Ann. Surg..

[62] Sabater L., Ausania F., Bakker O.J., Boadas J., Domínguez-Muñoz J.E., Falconi M., Fernández-Cruz L., Frulloni L., González-Sánchez V., Lariño-Noia J. (2016). Evidence-based guidelines for the management of exocrine pancreatic insufficiency after pancreatic surgery. Ann. Surg..

[63] Crippa S., Bassi C., Warshaw A.L., Falconi M., Partelli S., Thayer S.P., Pederzoli P., Fernández-del Castillo C. (2007). Middle pancreatectomy: Indications, short- and long-term operative outcomes. Ann. Surg..

[64] Müller M.W., Friess H., Kleeff J., Hinz U., Wente M.N., Paramythiotis D., Berberat P.O., Ceyhan G.O., Büchler M.W. (2006). Middle segmental pancreatic resection: An option to treat benign pancreatic body lesions. Ann. Surg..

[65] Paiella S., De Pastena M., Faustini F., Landoni L., Pollini T., Bonamini D., Giuliani T., Bassi C., Esposito A., Tuveri M. (2018). Central pancreatectomy for benign or low-grade malignant pancreatic lesions-A single-center retrospective analysis of 116 cases. Eur. J. Surg. Oncol. (EJSO).

[66] Falconi M., Mantovani W., Crippa S., Mascetta G., Salvia R., Pederzoli P. (2007). Pancreatic insufficiency after different resections for benign tumours. BJS.

[67] Jilesen A.P.J., van Eijck C.H.J., Busch O.R.C., van Gulik T.M., Gouma D.J., van Dijkum E.J.M.N. (2016). Postoperative outcomes of enucleation and standard resections in patients with a pancreatic neuroendocrine tumor. World J. Surg..

[68] DiNorcia J., Lee M.K., Reavey P.L., Genkinger J.M., Lee J.A., Schrope B.A., Chabot J.A., Allendorf J.D. (2010). One Hundred Thirty Resections for Pancreatic Neuroendocrine Tumor: Evaluating the Impact of Minimally Invasive and Parenchyma-Sparing Techniques. J. Gastrointest. Surg..

[69] Falconi M., Zerbi A., Crippa S., Balzano G., Boninsegna L., Capitanio V., Bassi C., Di Carlo V., Pederzoli P. (2010). Parenchyma-Preserving Resections for Small Nonfunctioning Pancreatic Endocrine Tumors. Ann. Surg. Oncol..

[70] Bettini R., Boninsegna L., Mantovani W., Capelli P., Bassi C., Pederzoli P., Fave G.F.D., Panzuto F., Scarpa A., Falconi M. (2008). Prognostic factors at diagnosis and value of WHO classification in a mono-institutional series of 180 non-functioning pancreatic endocrine tumours. Ann. Oncol..

[71] Tomassetti P., Campana D., Piscitelli L., Casadei R., Santini D., Nori F., Morselli-Labate A.M., Pezzilli R., Corinaldesi R. (2005). Endocrine pancreatic tumors: Factors correlated with survival. Ann. Oncol..

[72] Hashim Y.M., Trinkaus K.M., Linehan D.C., Strasberg S.S., Fields R.C., Cao D., Hawkins W.G. (2014). Regional Lymphadenectomy Is Indicated in the Surgical Treatment of Pancreatic Neuroendocrine Tumors (PNETs). Ann. Surg..

[73] Zhang Z., Liu M., Ji S., Luo G., Xu W., Liu W., Hu Q., Sun Q., Ye Z., Qin Y. (2020). Prognostic Value and Clinical Predictors of Lymph Node Metastases in Pancreatic Neuroendocrine Tumors. Pancreas.

[74] Curran T., Pockaj B.A., Gray R.J., Halfdanarson T., Wasif N. (2014). Importance of Lymph Node Involvement in Pancreatic Neuroendocrine Tumors: Impact on Survival and Implications for Surgical Resection. J. Gastrointest. Surg..

[75] Mao R., Zhao H., Li K., Luo S., Turner M., Cai J.-Q., Blazer D. (2019). Outcomes of Lymph Node Dissection for Non-metastatic Pancreatic Neuroendocrine Tumors: A Propensity Score-Weighted Analysis of the National Cancer Database. Ann. Surg. Oncol..

[76] Berg K. (2018). Management of functional neuroendocrine tumors of the pancreas. Gland Surg..

[77] Knigge U., Hansen C.P. (2012). Surgery for GEP-NETs. Best Pract. Res. Clin. Gastroenterol..

[78] Nikfarjam M., Warshaw A.L., Axelrod L., Deshpande V., Thayer S.P., Ferrone C.R., Fernández-del Castillo C. (2008). Improved contemporary surgical management of insulinomas: A 25-year experience at the Massachusetts General Hospital. Ann. Surg..

[79] Mehrabi A., Fischer L., Hafezi M., Dirlewanger A., Grenacher L., Diener M.K., Fonouni H., Golriz M., Garoussi C., Fard N. (2014). A Systematic Review of Localization, Surgical Treatment Options, and Outcome of Insulinoma. Pancreas.

[80] Al-Kurd A., Chapchay K., Grozinsky-Glasberg S., Mazeh H. (2014). Laparoscopic resection of pancreatic neuroendocrine tumors. World J. Gastroenterol..

[81] Belfiori G., Wiese D., Partelli S., Wächter S., Maurer E., Crippa S., Falconi M., Bartsch D.K. (2018). Minimally Invasive Versus Open Treatment for Benign Sporadic Insulinoma Comparison of Short-Term and Long-Term Outcomes. World J. Surg..

[82] Xu J., Li F., Zhan H., Liu H., Wu D., Hu S., Wang L. (2020). Laparoscopic enucleation of pancreatic tumours: A single-institution experience of 66 cases. ANZ J. Surg..

[83] Su A.-P., Ke N.-W., Zhang Y., Liu X.-B., Hu W.-M., Tian B.-L., Zhang Z.-D. (2014). Is laparoscopic approach for pancreatic insulinomas safe? Results of a systematic review and meta-analysis. J. Surg. Res..

[84] Furnica R.M., Deprez P., Maiter D., Vandeleene B., Borbath I. (2020). Endoscopic ultrasound-guided radiofrequency ablation: An effective and safe alternative for the treatment of benign insulinoma. Ann. d’Endocrinol..

[85] Rimbaş M., Horumbă M., Rizzatti G., Crinò S.F., Gasbarrini A., Costamagna G., Larghis A. (2020). Interventional endoscopic ultrasound for pancreatic neuroendocrine neoplasms. Dig. Endosc..

[86] Yuan T., Liu S., Zhu C., Dong Y., Zhu H., Wu X., Tang Y., Zhao W. (2021). Continuous Glucose Monitoring in Patients With Insulinoma Treated by Endoscopic Ultrasound-Guided Ethanol Injection. Pancreas.

[87] Akca A., Starke A.A.R., Dobek A., Ulrich A., Goretzki P.E. (2019). Early Postoperative Fasting Serum Glucose Levels are Useful in Depicting Future Diabetes Mellitus in Patients with Curative Insulinoma Surgery. Exp. Clin. Endocrinol. Diabetes.

[88] Hirschowitz B.I., Mohnen J., Shaw S. (1996). Long-term treatment with lansoprazole for patients with Zollinger-Ellison syndrome. Aliment. Pharmacol. Ther..

[89] Norton J.A., Jensen R.T. (2004). Resolved and Unresolved Controversies in the Surgical Management of Patients with Zollinger-Ellison Syndrome. Ann. Surg..

[90] Ojeaburu J.V., Ito T., Crafa P., Bordi C., Jensen R.T. (2010). Mechanism of Acid Hypersecretion Post Curative Gastrinoma Resection. Dig. Dis. Sci..

[91] Norton J.A., Foster D.S., Ito T., Jensen R.T. (2018). Gastrinomas: Medical or surgical treatment. Endocrinol. Metab. Clin. N. Am..

[92] Norton J.A., Fraker D.L., Alexander H.R., Gibril F., Liewehr D.J., Venzon D.J., Jensen R.T. (2006). Surgery Increases Survival in Patients with Gastrinoma. Ann. Surg..

[93] Norton J.A., Fraker D.L., Alexander H.R., Jensen R.T. (2012). Value of Surgery in Patients with Negative Imaging and Sporadic Zollinger-Ellison Syndrome. Ann. Surg..

[94] Bartsch D.K., Waldmann J., Fendrich V., Boninsegna L., Lopez C.L., Partelli S., Falconi M. (2012). Impact of lymphadenectomy on survival after surgery for sporadic gastrinoma. BJS.

[95] Larsson C., Skogseid B., Öberg K., Nakamura Y., Nordenskjöld M. (1988). Multiple endocrine neoplasia type 1 gene maps to chromosome 11 and is lost in insulinoma. Nature.

[96] Brandi M.L., Gagel R.F., Angeli A., Bilezikian J.P., Beck-Peccoz P., Bordi C., Conte-Devolx B., Falchetti A., Gheri R.G., Libroia A. (2001). Guidelines for diagnosis and therapy of MEN type 1 and type 2. J. Clin. Endocrinol. Metab..

[97] Tonelli F., Giudici F., Giusti F., Brandi M.L. (2012). Gastroenteropancreatic Neuroendocrine Tumors in Multiple Endocrine Neoplasia Type 1. Cancers.

[98] Ito T., Igarashi H., Jensen R.T. (2012). Pancreatic neuroendocrine tumors: Clinical features, diagnosis and medical treatment: Advances. Best Pract. Res. Clin. Gastroenterol..

[99] Lopez C.L., Waldmann J., Fendrich V., Langer P., Kann P.H., Bartsch D.K. (2011). Long-term results of surgery for pancreatic neuroendocrine neoplasms in patients with MEN1. Langenbecks Arch. Surg..

[100] Triponez F., Dosseh D., Goudet P., Cougard P., Bauters C., Murat A., Cadiot G., Niccoli-Sire P., Chayvialle J.-A., Calender A. (2006). Epidemiology Data on 108 MEN 1 Patients from the GTE With Isolated Nonfunctioning Tumors of the Pancreas. Ann. Surg..

[101] Partelli S., Tamburrino D., Lopez C., Albers M., Milanetto A.C., Pasquali C., Manzoni M., Toumpanakis C., Fusai G., Bartsch D. (2016). Active Surveillance versus Surgery of Nonfunctioning Pancreatic Neuroendocrine Neoplasms ≤2 cm in MEN1 Patients. Neuroendocrinology.

[102] Triponez F., Goudet P., Dosseh D., Cougard P., Bauters C., Murat A., Cadiot G., Niccoli-Sire P., Calender A., Proye C.A.G. (2006). Is Surgery Beneficial for MEN1 Patients with Small (≤2 cm), Nonfunctioning Pancreaticoduodenal Endocrine Tumor? An Analysis of 65 Patients from the GTE. World J. Surg..

[103] La Salvia A., Sesti F., Grinzato C., Mazzilli R., Tarsitano M.G., Giannetta E., Faggiano A. (2021). Somatostatin analogue therapy in MEN1-related pancreatic neuroendocrine tumors from evidence to clinical practice: A systematic review. Pharmaceuticals.

[104] Triponez F., Sadowski S.M., Pattou F., Cardot-Bauters C., Mirallié E., Le Bras M., Sebag F., Niccoli P., Deguelte S., Cadiot G. (2018). Long-term Follow-up of MEN1 Patients Who Do Not Have Initial Surgery for Small ≤2 cm Nonfunctioning Pancreatic Neuroendocrine Tumors, an AFCE and GTE Study: Association Francophone de Chirurgie Endocrinienne & Groupe d’Etude des Tumeurs Endocrines. Ann. Surg..

[105] Niederle B., Selberherr A., Bartsch D.K., Brandi M.L., Doherty G.M., Falconi M., Goudet P., Halfdanarson T.R., Ito T., Jensen R.T. (2020). Multiple Endocrine Neoplasia Type 1 (MEN1) and the Pancreas-Diagnosis and Treatment of Functioning and Non-Functioning Pancreatic and Duodenal Neuroendocrine Neoplasia within the MEN1 Syndrome–An International Consensus Statement. Neuroendocrinology.

[106] Albers M.B., Manoharan J., Bollmann C., Chlosta M.P., Holzer K., Bartsch D.K. (2018). Results of Duodenopancreatic Reoperations in Multiple Endocrine Neoplasia Type 1. World J. Surg..

[107] Halfdanarson T.R., Rubin J., Farnell M.B., Grant C.S., Petersen G.M. (2008). Pancreatic endocrine neoplasms: Epidemiology and prognosis of pancreatic endocrine tumors. Endocr.-Relat. Cancer.

[108] Bartsch D.K., Albers M., Knoop R.F., Kann P.H., Fendrich V., Waldmann J. (2013). Enucleation and Limited Pancreatic Resection Provide Long-Term Cure for Insulinoma in Multiple Endocrine Neoplasia Type 1. Neuroendocrinology.

[109] Giudici F., Nesi G., Brandi M.L., Tonelli F. (2012). Surgical Management of Insulinomas in Multiple Endocrine Neoplasia Type 1. Pancreas.

[110] Van Beek D.J., Nell S., Verkooijen H.M., Rinkes I.H.M.B., Valk G.D., Vriens M.R., Goudet P., Vella A., Donegan D., Bartsch D.K. (2020). Surgery for multiple endocrine neoplasia type 1-related insulinoma: Long-term outcomes in a large international cohort. BJS.

[111] Vezzosi D., Cardot-Bauters C., Bouscaren N., Lebras M., Bertholon-Grégoire M., Niccoli P., Levy-Bohbot N., Groussin L., Bouchard P., Tabarin A. (2015). Long-term results of the surgical management of insulinoma patients with MEN1: A Groupe d’étude des Tumeurs Endocrines (GTE) retrospective study. Eur. J. Endocrinol..

[112] Anlauf M., Garbrecht N., Henopp T., Schmitt A., Schlenger R., Raffel A., Krausch M., Gimm O., Eisenberger C.F., Knoefel W.T. (2006). Sporadic versus hereditary gastrinomas of the duodenum and pancreas: Distinct clinico-pathological and epidemiological features. World J. Gastroenterol..

[113] Guarnotta V., Martini C., Davì M.V., Pizza G., Colao A., Faggiano A., NIKE group (2018). The Zollinger-Ellison syndrome: Is there a role for somatostatin analogues in the treatment of the gastrinoma?. Endocrine.

[114] Lopez C.L., Falconi M., Waldmann J., Boninsegna L., Fendrich V., Goretzki P.K., Langer P., Kann P.H., Partelli S., Bartsch D.K. (2013). Partial Pancreaticoduodenectomy Can Provide Cure for Duodenal Gastrinoma Associated with Multiple Endocrine Neoplasia Type 1. Ann. Surg..

[115] Norton J.A., Alexander H.R., Fraker D.L., Venzon D.J., Gibril F., Jensen R.T. (2001). Comparison of Surgical Results in Patients with Advanced and Limited Disease With Multiple Endocrine Neoplasia Type 1 and Zollinger-Ellison Syndrome. Ann. Surg..

[116] Alrezk R., Hannah-Shmouni F., Stratakis C.A. (2017). MEN4 and CDKN1B mutations: The latest of the MEN syndromes. Endocr.-Relat. Cancer.

[117] Lonser R.R., Glenn G.M., Walther M., Chew E.Y., Libutti S.K., Linehan W.M., Oldfield E.H. (2003). Von Hippel-Lindau disease. Lancet.

[118] Keutgen X.M., Hammel P., Choyke P.L., Libutti S.K., Jonasch E., Kebebew X.M.K.E. (2016). Evaluation and management of pancreatic lesions in patients with von Hippel–Lindau disease. Nat. Rev. Clin. Oncol..

[119] Igarashi H., Ito T., Nishimori I., Tamura K., Yamasaki I., Tanaka M., Shuin T. (2013). Pancreatic involvement in Japanese patients with von Hippel-Lindau disease: Results of a nationwide survey. J. Gastroenterol..

[120] Weisbrod A.B., Kitano M., Thomas F., Williams D., Gulati N., Gesuwan K., Liu Y., Venzon D., Turkbey I., Choyke P. (2013). Assessment of Tumor Growth in Pancreatic Neuroendocrine Tumors in von Hippel Lindau Syndrome. J. Am. Coll. Surg..

[121] Blansfield J.A., Choyke L., Morita S.Y., Choyke P.L., Pingpank J.F., Alexander H.R., Seidel G., Shutack Y., Yuldasheva N., Eugeni M. (2007). Clinical, genetic and radiographic analysis of 108 patients with von Hippel-Lindau disease (VHL) manifested by pancreatic neuroendocrine neoplasms (PNETs). Surgery.

[122] Penitenti F., Landoni L., Scardoni M., Piredda M.L., Cingarlini S., Scarpa A., D’Onofrio M., Girelli D., Davi M.V. (2021). Clinical presentation, genotype-phenotype correlations, and outcome of pancreatic neuroendocrine tumors in Von Hippel-Lindau syndrome. Endocrine.

[123] Libutti S.K., Choyke P.L., Alexander H., Glenn G., Bartlett D.L., Zbar B., Lubensky I., McKee S.A., Maher E.R., Linehan W. (2000). Clinical and genetic analysis of patients with pancreatic neuroendocrine tumors associated with von Hippel-Lindau disease. Surgery.

[124] Hammel P.R., Vilgrain V., Terris B., Penfornis A., Sauvanet A., Correas J.M., Chauveau D., Balian A., Beigelman C., O’Toole D. (2000). Pancreatic involvement in von Hippel-Lindau disease. The Groupe Francophone d’Etude de la Maladie de von Hippel-Lindau. Gastroenterology.

[125] De Mestier L., Gaujoux S., Cros J., Hentic O., Vullierme M.-P., Couvelard A., Cadiot G., Sauvanet A., Ruszniewski P., Richard S. (2015). Long-term prognosis of resected pancreatic neuroendocrine tumors in von hippel-lindau disease is favorable and not influenced by small tumors left in place. Ann. Surg..

[126] Gutmann D.H., Ferner R.E., Listernick R.H., Korf B.R., Wolters P.L., Johnson K.J. (2017). Neurofibromatosis 1. Nat. Rev. Dis. Primers.

[127] Mao C., Shah A., Hanson D.J., Howard J.M. (1995). Von recklinghausen’s disease associated with duodenal somatostatinoma: Contrast of duodenal versus pancreatic somatostatinomas. J. Surg. Oncol..

[128] Nishi T., Kawabata Y., Hari Y., Imaoka H., Ishikawa N., Yano S., Maruyama R., Tajima Y. (2012). A case of pancreatic neuroendocrine tumor in a patient with neurofibromatosis-1. World J. Surg. Oncol..

[129] Relles D., Baek J., Witkiewicz A., Yeo C.J. (2010). Periampullary and Duodenal Neoplasms in Neurofibromatosis Type 1: Two Cases and an Updated 20-Year Review of the Literature Yielding 76 Cases. J. Gastrointest. Surg..

[130] Clements W.M., Martin S.P., Stemmerman G., Lowy A.M. (2003). Ampullary carcinoid tumors: Rationale for an aggressive surgical approach. J. Gastrointest. Surg..

[131] Hartel M., Wente M.N., Sido B., Friess H., Buchler M.W. (2005). Carcinoid of the ampulla of Vater. J. Gastroenterol. Hepatol..

[132] Sandru F., Carsote M., Valea A., Albu S.E., Petca R.-C., Dumitrascu M.C. (2020). Somatostatinoma: Beyond neurofibromatosis type 1 (Review). Exp. Ther. Med..

[133] Rasmussen S.A., Yang Q., Friedman J. (2001). Mortality in Neurofibromatosis 1: An Analysis Using U.S. Death Certificates. Am. J. Hum. Genet..

[134] Crino P.B., Nathanson K.L., Henske E.P. (2006). The tuberous sclerosis complex. N. Engl. J. Med..

[135] Müller S.A., Hartel M., Mehrabi A., Welsch T., Martin D.J., Hinz U., Schmied B.M., Büchler M.W. (2009). Vascular Resection in Pancreatic Cancer Surgery: Survival Determinants. J. Gastrointest. Surg..

[136] Zhou Y., Zhang Z., Liu Y., Li B., Xu D. (2012). Pancreatectomy Combined with Superior Mesenteric Vein–Portal Vein Resection for Pancreatic Cancer: A Meta-analysis. World J. Surg..

[137] Belfiori G., Fiorentini G., Tamburrino D., Partelli S., Pagnanelli M., Gasparini G., Castoldi R., Balzano G., Rubini C., Zamboni G. (2021). Vascular resection during pancreatectomy for pancreatic head cancer: A technical issue or a prognostic sign?. Surgery.

[138] Shimada K., Sano T., Sakamoto Y., Kosuge T. (2006). Clinical Implications of Combined Portal Vein Resection as a Palliative Procedure in Patients Undergoing Pancreaticoduodenectomy for Pancreatic Head Carcinoma. Ann. Surg. Oncol..

[139] Norton J.A., Harris E.J., Chen Y., Visser B.C., Poultsides G.A., Kunz P.C., Fisher G.A., Jensen R.T. (2011). Pancreatic Endocrine Tumors with Major Vascular Abutment, Involvement, or Encasement and Indication for Resection. Arch. Surg..

[140] Haugvik S.-P., Labori K.J., Waage A., Line P.-D., Mathisen Ø., Gladhaug I.P. (2013). Pancreatic Surgery with Vascular Reconstruction in Patients with Locally Advanced Pancreatic Neuroendocrine Tumors. J. Gastrointest. Surg..

[141] Birnbaum D.J., Turrini O., Vigano L., Russolillo N., Autret A., Moutardier V., Capussotti L., Le Treut Y.-P., Delpero J.-R., Hardwigsen J. (2014). Surgical Management of Advanced Pancreatic Neuroendocrine Tumors: Short-Term and Long-Term Results from an International Multi-institutional Study. Ann. Surg. Oncol..

[142] Thiels C.A., Bergquist J.R., Laan D.V., Croome K.P., Smoot R.L., Nagorney D.M., Thompson G.B., Kendrick M.L., Farnell M.B., Truty M.J. (2016). Outcomes of pancreaticoduodenectomy for pancreatic neuroendocrine tumors: Are combined procedures justified?. J. Gastrointest. Surg..

[143] Titan A.L., Norton J.A., Fisher A.T., Foster D.S., Harris E.J., Worhunsky D.J., Worth P.J., Dua M.M., Visser B.C., Poultsides G.A. (2020). Evaluation of Outcomes Following Surgery for Locally Advanced Pancreatic Neuroendocrine Tumors. JAMA Netw. Open.

[144] Teh S.H., Deveney C., Sheppard B.C. (2007). Aggressive pancreatic resection for primary pancreatic neuroendocrine tumor: Is it justifiable?. Am. J. Surg..

[145] Xie H., Liu J., Yadav S., Keutgen X.M., Hobday T.J., Strosberg J.R., Halfdanarson T.R. (2019). The Role of Perioperative Systemic Therapy in Localized Pancreatic Neuroendocrine Neoplasms. Neuroendocrinology.

[146] Sowa-Staszczak A., Pach D., Chrzan R., Trofimiuk M., Stefańska A., Tomaszuk M., Kołodziej M., Mikołajczak R., Pawlak D., Hubalewska-Dydejczyk A. (2011). Peptide receptor radionuclide therapy as a potential tool for neoadjuvant therapy in patients with inoperable neuroendocrine tumours (NETs). Eur. J. Nucl. Med. Mol. Imaging.

[147] Ostwal V., Basu S., Bhargava P., Shah M., Parghane R.V., Srinivas S., Chaudhari V., Bhandare M.S., Shrikhande S.V., Ramaswamy A. (2020). Capecitabine-Temozolomide in Advanced Grade 2 and Grade 3 Neuroendocrine Neoplasms: Benefits of Chemotherapy in Neuroendocrine Neoplasms with Significant ^18^FDG Uptake. Neuroendocrinology.

[148] Ambe C., Nguyen P., Centeno B., Choi J., Strosberg J.R., Kvols L.K., Hodul P.J., Hoffe S.E., Malafa M.P. (2015). Multimodality management of borderline resectable pancreatic neuroendocrine tumors: Sentinel report of a single-institutional experience. J. Clin. Oncol..

[149] Franko J., Feng W., Yip L., Genovese E., Moser A.J. (2009). Non-functional Neuroendocrine Carcinoma of the Pancreas: Incidence, Tumor Biology, and Outcomes in 2,158 Patients. J. Gastrointest. Surg..

[150] Yao J.C., Eisner M.P., Leary C., Dagohoy C., Phan A., Rashid A., Hassan M., Evans D.B. (2007). Population-Based Study of Islet Cell Carcinoma. Ann. Surg. Oncol..

[151] Yao J.C., Pavel M., Lombard-Bohas C., Van Cutsem E., Voi M., Brandt U., Catherine L.-B., Chen D., Capdevila J., de Vries E. (2016). Everolimus for the Treatment of Advanced Pancreatic Neuroendocrine Tumors: Overall Survival and Circulating Biomarkers from the Randomized, Phase III RADIANT-3 Study. J. Clin. Oncol..

[152] Faivre S., Niccoli P., Castellano D., Valle J.W., Hammel P., Raoul J.-L., Vinik A., Van Cutsem E., Bang Y.-J., Lee S.-H. (2016). Sunitinib in pancreatic neuroendocrine tumors: Updated progression-free survival and final overall survival from a phase III randomized study. Ann. Oncol..

[153] Brabander T., Van Der Zwan W.A., Teunissen J.J., Kam B.L., Feelders R.A., De Herder W.W., Van Eijck C.H., Franssen G.J., Krenning E.P., Kwekkeboom D.J. (2017). Long-Term Efficacy, Survival, and Safety of [177Lu-DOTA0,Tyr3]octreotate in Patients with Gastroenteropancreatic and Bronchial Neuroendocrine Tumors. Clin. Cancer Res..

[154] Rossi R.E., Massironi S., Conte D., Peracchi M. (2014). Therapy for metastatic pancreatic neuroendocrine tumors. Ann. Transl. Med..

[155] Norton J.A., Warren R.S., Kelly M.G., Zuraek M.B., Jensen R.T. (2003). Aggressive surgery for metastatic liver neuroendocrine tumors. Surgery.

[156] Mayo S.C., de Jong M.C., Pulitano C., Clary B.M., Reddy S.K., Gamblin T.C., Celinksi S.A., Kooby D.A., Staley C.A., Stokes J.B. (2010). Surgical Management of Hepatic Neuroendocrine Tumor Metastasis: Results from an International Multi-Institutional Analysis. Ann. Surg. Oncol..

[157] Cusati D., Zhang L., Harmsen W.S., Hu A., Farnell M.B., Nagorney D.M., Donohue J.H., Que F.G., Reid-Lombardo K.M., Kendrick M.L. (2012). Metastatic Nonfunctioning Pancreatic Neuroendocrine Carcinoma to Liver: Surgical Treatment and Outcomes. J. Am. Coll. Surg..

[158] Sarmiento J.M., Heywood G., Rubin J., Ilstrup D.M., Nagorney D.M., Que F.G. (2003). Surgical treatment of neuroendocrine metastases to the liver: A plea for resection to increase survival. J. Am. Coll. Surg..

[159] Elias D., Lasser P., Ducreux M., Duvillard P., Ouellet J.-F., Dromain C., Schlumberger M., Pocard M., Boige V., Miquel C. (2003). Liver resection (and associated extrahepatic resections) for metastatic well-differentiated endocrine tumors: A 15-year single center prospective study. Surgery.

[160] Chawla A., Williams R.T., Sich N., Clancy T., Wang J., Ashley S., Pezzi C., Swanson R. (2018). Pancreaticoduodenectomy and metastasectomy for metastatic pancreatic neuroendocrine tumors. J. Surg. Oncol..

[161] Lania A., Ferraù F., Rubino M., Modica R., Colao A., Faggiano A. (2021). Neoadjuvant Therapy for Neuroendocrine Neoplasms: Recent Progresses and Future Approaches. Front. Endocrinol..

[162] Cloyd J.M., Omichi K., Mizuno T., Kawaguchi Y., Tzeng C.-W.D., Conrad C., Chun Y.S., Aloia T.A., Katz M.H.G., Lee J.E. (2018). Preoperative Fluorouracil, Doxorubicin, and Streptozocin for the Treatment of Pancreatic Neuroendocrine Liver Metastases. Ann. Surg. Oncol..

[163] Strosberg J.R., Fine R.L., Choi J., Nasir A., Coppola D., Chen D.T., Helm J., Kvols L. (2011). First-line chemotherapy with capecitabine and temozolomide in patients with metastatic pancreatic endocrine carcinomas. Cancer.

[164] Squires M.H., Worth P., Konda B., Shah M.H., Dillhoff M.E., Abdel-Misih S., Norton J.A., Visser B.C., Dua M., Pawlik T.M. (2020). Neoadjuvant Capecitabine/Temozolomide for Locally Advanced or Metastatic Pancreatic Neuroendocrine Tumors. Pancreas.

[165] Stoeltzing O., Loss M., Huber E., Gross V., Eilles C., Mueller-Brand J., Schlitt H.J. (2009). Staged surgery with neoadjuvant 90Y-DOTATOC therapy for down-sizing synchronous bilobular hepatic metastases from a neuroendocrine pancreatic tumor. Langenbeck’s Arch. Surg..

[166] Parghane R.V., Ostwal V., Ramaswamy A., Bhandare M., Chaudhari V., Talole S., Shrikhande S.V., Basu S. (2020). Long-term outcome of “Sandwich” chemo-PRRT: A novel treatment strategy for metastatic neuroendocrine tumors with both FDG- and SSTR-avid aggressive disease. Eur. J. Nucl. Med. Mol. Imaging.

[167] Frilling A., Li J., Malamutmann E., Schmid K., Bockisch A., Broelsch C.E. (2009). Treatment of liver metastases from neuroendocrine tumours in relation to the extent of hepatic disease. BJS.

[168] Elias M., Santoro R., Ouellet J.-F., Osmak L., De Baere T., Roche A. (2004). Simultaneous percutaneous right portal vein embolization and left liver tumor radiofrequency ablation prior to a major right hepatic resection for bilateral colorectal metastases. Hepatogastroenterology.

[169] Kianmanesh R., Farges O., Abdalla E.K., Sauvanet A., Ruszniewski P., Belghiti J. (2003). Right portal vein ligation: A new planned two-step all-surgical approach for complete resection of primary gastrointestinal tumors with multiple bilateral liver metastases. J. Am. Coll. Surg..

[170] Kianmanesh R., Sauvanet A., Hentic O., Couvelard A., Lévy P., Vilgrain V., Ruszniewski P., Belghiti J. (2008). Two-step Surgery for Synchronous Bilobar Liver Metastases from Digestive Endocrine Tumors: A Safe Approach for Radical Resection. Ann. Surg..

[171] Sandström P., Røsok B.I., Sparrelid E., Larsen P.N., Larsson A.L., Lindell G., Schultz N.A., Bjørnbeth B.A., Isaksson B., Rizell M. (2018). ALPPS improves resectability compared with conventional two-stage hepatectomy in patients with advanced colorectal liver metastasis: Results from a Scandinavian multicenter randomized controlled trial (LIGRO Trial). Ann. Surg..

[172] Linecker M., Kambakamba P., Raptis D.A., Malagó M., Ratti F., Aldrighetti L., Robles-Campos R., Lehwald-Tywuschik N., Knoefel W.T., Balci D. (2019). ALPPS in neuroendocrine liver metastases not amenable for conventional resection–lessons learned from an interim analysis of the International ALPPS Registry. HPB.

[173] Chambers A.J., Pasieka J.L., Dixon E., Rorstad O. (2008). The palliative benefit of aggressive surgical intervention for both hepatic and mesenteric metastases from neuroendocrine tumors. Surgery.

[174] Graff-Baker A.N., Sauer D.A., Pommier S.J., Pommier R.F. (2014). Expanded criteria for carcinoid liver debulking: Maintaining survival and increasing the number of eligible patients. Surgery.

[175] Maxwell J.E., Sherman S., O’Dorisio T., Bellizzi A., Howe J.R. (2015). Liver-directed surgery of neuroendocrine metastases: What is the optimal strategy?. Surgery.

[176] Morgan R.E., Pommier S.J., Pommier R.F. (2018). Expanded criteria for debulking of liver metastasis also apply to pancreatic neuroendocrine tumors. Surgery.

[177] Landry C.S., Scoggins C.R., McMasters K.M., Martin R.C. (2008). Management of hepatic metastasis of gastrointestinal carcinoid tumors. J. Surg. Oncol..

[178] Oberg K. (2010). Pancreatic endocrine tumors. Semin Oncol..

[179] Partelli S., Inama M., Tamburrino D., Caplin M.E., Rinke A., Begum N., Valente R., Fendrich V., Keck T., Bartsch D. (2015). Long-Term Outcomes of Surgical Management of Pancreatic Neuroendocrine Tumors with Synchronous Liver Metastases. Neuroendocrinology.

[180] Keutgen X.M., Nilubol N., Glanville J., Sadowski S.M., Liewehr D.J., Venzon D.J., Steinberg S.M., Kebebew E. (2015). Resection of primary tumor site is associated with prolonged survival in metastatic nonfunctioning pancreatic neuroendocrine tumors. Surgery.

[181] Bertani E., Fazio N., Botteri E., Chiappa A., Falconi M., Grana C., Bodei L., Papis D., Spada F., Bazolli B. (2014). Resection of the primary pancreatic neuroendocrine tumor in patients with unresectable liver metastases: Possible indications for a multimodal approach. Surgery.

[182] Bertani E., Fazio N., Radice D., Zardini C., Spinoglio G., Chiappa A., Ribero D., Biffi R., Partelli S., Falconi M. (2016). Assessing the role of primary tumour resection in patients with synchronous unresectable liver metastases from pancreatic neuroendocrine tumour of the body and tail. A propensity score survival evaluation. Eur. J. Surg. Oncol. (EJSO).

[183] Bertani E., Fazio N., Radice D., Zardini C., Grana C., Bodei L., Funicelli L., Ferrari C., Spada F., Partelli S. (2016). Resection of the Primary Tumor Followed by Peptide Receptor Radionuclide Therapy as Upfront Strategy for the Treatment of G1–G2 Pancreatic Neuroendocrine Tumors with Unresectable Liver Metastases. Ann. Surg. Oncol..

[184] Capurso G., Bettini R., Rinzivillo M., Boninsegna L., Fave G.D., Falconi M. (2011). Role of Resection of the Primary Pancreatic Neuroendocrine Tumour Only in Patients with Unresectable Metastatic Liver Disease: A Systematic Review. Neuroendocrinology.

[185] Shimata K., Sugawara Y., Hibi T. (2018). Liver transplantation for unresectable pancreatic neuroendocrine tumors with liver metastases in an era of transplant oncology. Gland. Surg..

[186] Le Treut Y.P., Grégoire E., Klempnauer J., Belghiti J., Jouve E., Lerut J., Castaing D., Soubrane O., Boillot O., Mantion G. (2013). For ELITA. Liver transplantation for neuroendocrine tumors in Europe-results and trends in patient selection: A 213-case European liver transplant registry study. Ann. Surg..

[187] Le Treut Y.P., Grégoire E., Belghiti J., Boillot O., Soubrane O., Mantion G., Cherqui D., Castaing D., Ruszniewski P., Wolf P. (2008). Predictors of long-term survival after liver transplantation for metastatic endocrine tumors: An 85-case French multicentric report. Am. J. Transplant..

[188] Van Vilsteren F.G., Baskin-Bey E.S., Nagorney D.M., Sanderson S.O., Kremers W.K., Rosen C.B., Gores G.J., Hobday T.J. (2006). Liver transplantation for gastroenteropancreatic neuroendocrine cancers: Defining selection criteria to improve survival. Liver Transpl..

[189] Máthé Z., Tagkalos E., Paul A., Molmenti E.P., Kóbori L., Fouzas I., Beckebaum S., Sotiropoulos G.C. (2011). Liver Transplantation for Hepatic Metastases of Neuroendocrine Pancreatic Tumors: A Survival-Based Analysis. Transplantation.

[190] Mazzaferro V., Pulvirenti A., Coppa J. (2007). Neuroendocrine tumors metastatic to the liver: How to select patients for liver transplantation?. J. Hepatol..

